# Teaching medical professionalism: a qualitative exploration of persuasive communication as an educational strategy

**DOI:** 10.1186/s12909-020-1993-0

**Published:** 2020-03-17

**Authors:** Michael Page, Paul Crampton, Rowena Viney, Antonia Rich, Ann Griffin

**Affiliations:** 1 Research Department of Medical Education, UCL Medical School, Royal Free Hospital, Room GF/664, London, NW3 2PF UK; 2 Hull York Medical School, York University, John Hughlings Jackson Building, University Rd, Heslington, York, YO10 5DD UK; 3 Research Department of Medical Education, UCL Medical School (UCLMS), The Directorate, 74 Huntley Street, London, WC1E 6AU UK

**Keywords:** Attitude change, Persuasive communication theory, Professionalism

## Abstract

**Background:**

Across the world, local standards provide doctors with a backbone of professional attitudes that must be embodied across their practice. However, educational approaches to develop attitudes are undermined by the lack of a theoretical framework. Our research explored the ways in which the General Medical Council’s (GMC) programme of preventative educational workshops (the Duties of a Doctor programme) attempted to influence doctors’ professional attitudes and examined how persuasive communication theory can advance understandings of professionalism education.

**Methods:**

This qualitative study comprised 15 ethnographic observations of the GMC’s programme of preventative educational workshops at seven locations across England, as well as qualitative interviews with 55 postgraduate doctors ranging in experience from junior trainees to senior consultants. The sample was purposefully chosen to include various geographic locations, different programme facilitators and doctors, who varied by seniority. Data collection occurred between March to December 2017. Thematic analysis was undertaken inductively, with meaning flowing from the data, and deductively, guided by persuasive communication theory.

**Results:**

The source (educator); the message (content); and the audience (participants) were revealed as key influences on the persuasiveness of the intervention. Educators established a high degree of credibility amongst doctors and worked to build rapport. Their message was persuasive, in that it drew on rational and emotional communicative techniques and made use of both statistical and narrative evidence. Importantly, the workshops were interactive, which allowed doctors to engage with the message and thus increased its persuasiveness.

**Conclusions:**

This study extends the literature by providing a theoretically-informed understanding of an educational intervention aimed at promoting professionalism, examining it through the lens of persuasive communication. Within the context of interactive programmes that allow doctors to discuss real life examples of professional dilemmas, educators can impact on doctors’ professional attitudes by drawing on persuasive communication techniques to enhance their credibility to demonstrate expertise, by building rapport and by making use of rational and emotional appeals.

## Background

Across the world, doctors are required to meet professional standards set by medical regulators [1–4] that provide doctors with a backbone of professional attitudes that must be embodied across their practice. While medical professionalism has been defined in a multiplicity of ways, many authors identify a behavioural component [5–7] and these behaviours are understood to be underpinned by attitudes [8–11]. This is in keeping with research from social psychology, which has established attitude formation as a critical component of behaviour [12], even if the relationship between the two can at times be complex [13].

It has been acknowledged that teaching professionalism is often challenging, with educational interventions at times being frustrated by the complexities present in the professional environment [14, 15]. Furthermore, some authors have pointed to the socially constructed, evolving nature of medical professionalism [16]. Consequently, there is flexibility needed in applying guidance to complex, emergent professional issues. However, despite agreement in the literature that professionalism should be addressed within medical education, there is widespread disagreement about *how* this should be done [17–21].

Against this backdrop, Birden et al. [22] undertook a systematic review in order to identify a unifying theoretical model of professionalism education, but concluded that none could be found. Authors such as Martin et al. [23] have identified the strongly cognitive and/or behavioural content of many classroom-based approaches and have drawn attention to the need for further research into how professional attitudes may be more effectively taught – as they point out, the settings in which doctors practise are value-laden cultural communities, and professionalism education should therefore take account of the strong influence of these contexts on individuals’ professional attitudes.

Given the largely atheoretical treatment of professionalism education in the literature to date, we drew on persuasive communication theory (PCT) to conceptualise how educators could influence doctors’ professional attitudes through a classroom-based educational intervention. Based on social psychological research, persuasive communication is defined as “any message that is intended to shape, reinforce or change the responses of another, or others” [24]. The premise is that certain approaches to communication are capable of changing attitudes, which are in turn linked to behaviour change [13]. There is empirical support for persuasive communication functioning in this way: within healthcare, there has been a particular focus on PCT in public health [25] and examples of where this has been shown to be effective include HIV and smoking cessation campaigns [26, 27].

Despite established links between PCT and attitudinal change, the application of PCT within professionalism education in medicine has remained absent from the literature. Consequently, we chose PCT as an established model for understanding the links between communication, attitude formation or attitude change and behavioural change amongst doctors. Importantly, and as described below, PCT provides a framework for systematically analysing several key components of communicative acts: the source of the communication; the message; and the audience.

The source (or educator) is important in changing attitudes, particularly by establishing their credibility [28] which is an audience’s subjective perception of the source’s expertise (‘is the educator a reputable source of knowledge or not?’) and trustworthiness (‘is the educator open and honest? Are they presenting a biased perspective?’). Source similarity is also key to persuasion, for example, holding similar memberships (e.g. belonging to the same organisation) and/or similar attitudes and beliefs.

The message (the content) includes the content of the intervention but also refers to specific features of delivery known to influence attitudes. There are two main types of appeal: rational and emotional. Firstly, rational appeals are those that present a logical argument supported with evidence. Quantitative evidence (e.g. statistics) tends to engage cognitive processing and is reported to influence attitudes, whilst qualitative evidence (e.g. narrative), is processed affectively and has a stronger impact on behavioural intention [29, 30]. Secondly, emotional appeals provoke affective responses, the use of fear or guilt to cause attitude change have been the most researched [12]. Appeals that strongly influence attitude change are those with a clear, convincing message. Emotional appeals that focus too heavily on fear can generate anxiety which undermines attitudinal change [31].

The audience (or participants) is the third salient aspect of PCT - both the educator and the message are evaluated by the audience [12]. ‘Message discrepancy’ describes a misalignment between the attitudes or knowledge currently held by an audience compared with the message the educator is trying to convey. Attitudinal change tends to happen when there is a moderate level of message discrepancy – change may be less likely when there is very little, or a great deal of, discrepancy [1, 32, 33]. Furthermore, active involvement with material that has been tailored to the audience and which is therefore personally relevant has also been shown to be persuasive [34].

This study set out to explore how to teach professionalism by applying a theoretical model from social psychology: PCT.

### The aim of the research

The aim of the study was to use PCT to explore the utility of persuasive communication in shaping professional attitudes during a programme of preventative educational workshops for qualified doctors. Specifically, our overarching research question was: how can PCT deepen our understanding of how to teach professionalism and shape professional attitudes?

Our objectives were:

- To identify the approaches to communication employed by workshop facilitators (the source) on the GMC’s Duties of a Doctor workshops;
- To analyse the features of the workshop content that contributed to the persuasiveness of the message;
- To explore qualitatively the ways in which the educational intervention had been successful in shaping doctors’ (i.e. the participants’) attitudes to professionalism;
- To make research-informed recommendations as to how classroom-based professionalism interventions can draw on persuasive communication techniques in order to enhance their effectiveness.

## Methods

### Setting - the GMC duties of a doctor programme

In recent years, medical regulators have expressed concern about the potential impact on doctors’ ability to maintain high professionalism standards throughout their careers [35]. In the UK, the General Medical Council’s (GMC) Duties of a Doctor preventative education programme is intended to influence doctors’ behaviours by providing guidance on the regulator’s standards and how they should be applied to professional conduct in a UK context, particularly in novel or ambiguous situations [36]. The programme consists of an educational outreach programme run at hospitals across England. The programme typically consists of five or six half-day workshops run over the course of 4 to 6 months. The content is tailored to the requirements of the specific cohort of doctors attending, but educators draw on a bank of standardised resources to ensure a degree of consistency in their teaching materials. Workshops are facilitated by members of the GMC’s Regional Liaison Service, known as Regional Liaison Advisors.

**Fig. 1:**
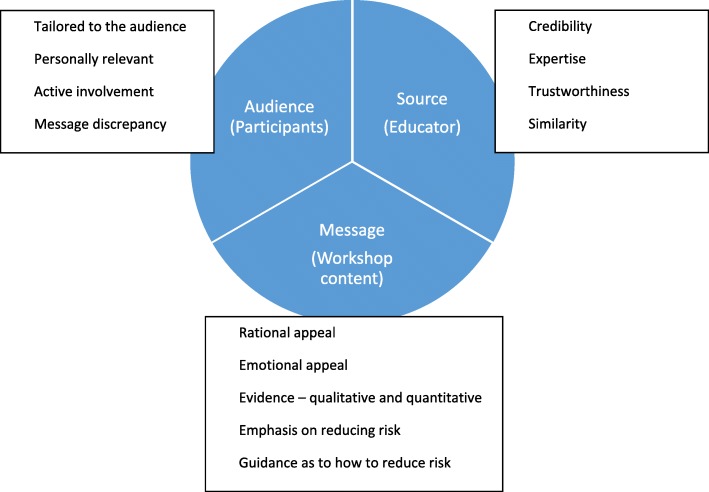
Three key components of persuasive communication theory and their constituent elements

In our research, PCT informed the analysis of interview and observational data drawn from the General Medical Council’s (GMC) programme of preventative educational workshops (Duties of a Doctor programme). We drew on the three key components in PCT: ‘the message’ that is the content of the educational intervention; ‘the source’ - in our case the educator; and ‘the audience’ i.e. the programme participants [12]. According to PCT, each of these three components comprises several constituent elements (see Fig. 1), which are explored in more detail below.

### Data collection

We conducted a qualitative, multi-methods study using detailed ethnographic observations of educator and participant interactions during the regulator’s professionalism programme as well as focus group interviews with workshop participants. Ethnography is an exploratory approach to research that typically involves analysis of unstructured data – data that is uncoded at the point of collection - and facilitates detailed observation of, and insights into, the cultures, practices and perspectives of a community [37]. Thus, it allowed us to explore the complex links between communication, attitudes and behaviour in the context of professionalism workshops for doctors. Ethics approval was granted by the University College London Ethics Committee (ref: 5490/001).

The workshops observed were selected purposively, focusing on a maximum variation sample in order to ensure inclusion of workshop topics and participant types (foundation doctors, staff grade doctors, consultants and general practitioners), including sampling from sessions at the beginning, middle and end of programmes. This approach also resulted in broad coverage of geographical regions (seven of the regulator’s thirteen English regions) and workshop facilitators (seven of the thirteen workshop educators),(Table 1). Programmes were excluded if they were outside the research timeline. Observations ran from March to December 2017, and researchers observed fifteen individual workshops at seven different hospital sites across the UK. In keeping with an ethnographic approach, which values the collection and interpretation of unstructured data, observation sheets were open-ended, with researchers making contemporaneous notes. A minimum of two members of the research team attended a sample of the workshops resulting in multiple sets of notes for each workshop observed.

**Table 1:** Details of the observed professionalism workshop sessions

Observation site	Cohort	Session no.	No. of attendees	Session topic(s)
Site 1	Established consultants and Senior trainees	6th (final)	8	Confidentiality
Site 2	Specialty doctors	3rd	7	Raising Concerns, Consent, Duty of Candour and Confidentiality
		5th (final)	3	Leadership and Management
Site 3	Foundation doctors	3rd	15	Complaints, Duty of Candour, Raising Concerns
Site 4	Consultants/Specialty doctors	1st	8	Identifying Learning Needs, Professional Boundaries, Personal Beliefs, Social Media
		2nd	9	Raising Concerns, Duty of Candour, Leadership and Management
		3rd	10	Confidentiality
Site 5	Consultants/Specialty doctors	3rd	6	Identifying, raising and acting on concerns, duty of candour and the role of apologies
		5th (final)	7	Leadership and Management
Site 6	General Practitioners/Consultants	1st	5	Identifying Learning Needs, Staying out of Trouble
		1st	6	Identifying Learning Needs, Staying out of Trouble
		2nd	7	Confidentiality
		2nd	5	Confidentiality
Site 7	New consultants	1st	6	Identifying Learning Needs, Complaints
		2nd	6	Leadership, Reflection, Confidentiality

Ten focus group interviews were conducted with 55 doctors from different locations, with interviewers following a semi-structured interview guide. The interview durations ranged from 34 to 59 min (mean duration 39 min) and were audio-recorded and professionally transcribed prior to analysis.

### Data analysis

**Fig. 2:**
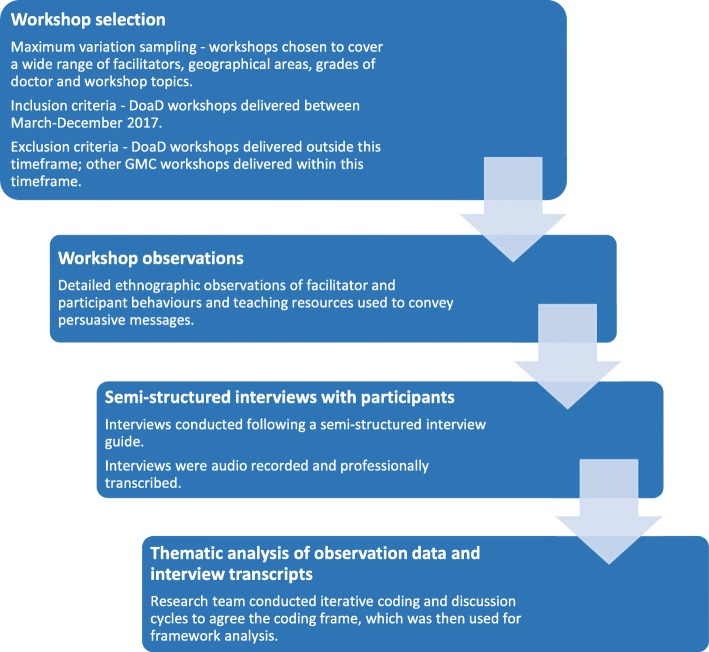
Flow diagram of the research process

The observation and interview data were imported into QSR International’s NVivo (Version 11) and a coding framework was developed inductively, based on open coding of the data, and deductively, drawing upon PCT [38]. Members of the research team initially undertook independent coding of a small sample of observation data and interview transcripts. Researchers then met to discuss the coding frameworks. Amendments were made and further rounds of coding and discussion were undertaken until the frameworks were agreed. The remainder of the data were then coded by the researchers (Fig. 2).

## Results

The section that follows presents the analysis of the observation and interview data. Observation data covered the full range of the GMC’s professionalism programme topics, type of delegate (consultant, trainee and speciality doctors) and stage of the programme (beginning, middle and end) (Table 1). Alongside the observations, 55 doctors drawn from all seven regions took part in focus group interviews between April and July 2017 (Table 2).

**Table 2:** Participant demographics for focus group interviews

Site	Group	Gender		Ethnicity				Total
		Male	Female	White/ White British	Asian/ Asian British	Other	Not given	
1	Established consultants and Senior trainees	1	2	3	0	0	0	3
1	Established consultants and Senior trainees	1	1	2	0	0	0	2
2	Specialty doctors	3	0	0	3	0	0	3
2	Specialty doctors (all of whom were international medical graduates)	3	0	0	3	0	0	3
3	Foundation doctors	8	8	8	3	0	5	16
3	Foundation doctors	3	5	5	1	0	2	8
4	Consultant/Specialty doctors	1	3	1	3	0	0	4
5	Consultants/Specialty doctors	3	5	6	2	0	0	8
6	General Practitioners/Consultants	3	0	1	1	1	0	3
7	New consultants	3	2	2	0	0	3	5
	Total	29	26	28	16	1	10	**55**

Findings from our analysis of the data are presented under the three main themes of the coding framework: the source (educator); the message (content); and the audience (participants).

### The source (educator)

Credibility was demonstrated principally through educator expertise. Educators showed a great deal of knowledge about the GMC’s professionalism guidance, frequently referring to specific paragraphs throughout the sessions. Educators also demonstrated their knowledge of the current context in which doctors are working and bolstered their breadth of real-world expertise by providing examples drawn from other hospitals across the region.

I think the person who leads [the workshop] and the way they do it will probably make a big difference….[the educator] was personable, he was relaxed, he was interesting and he was interested, I would say*, and he knew his bits and pieces…*I thought he was good, but I think the others did too, from talking to them...

Consultant-Senior trainee/UK graduate/Site 1 (our emphasis)

We observed educators attempting to establish their trustworthiness with the doctors by establishing source similarity. This was often done by emphasising certain likenesses with the doctors, despite not being doctors themselves. For example, one educator provided examples about their experiences “on the front line” in a different public-facing profession (Observation, Site 1). Another educator had experience of working in the hospital where the course was held and was able to demonstrate knowledge of the group’s working context (Observation, Site 4).

To broker a lack of source credibility, educators clearly articulated the clinical expertise of the doctors (audience) in the group by acknowledging their status. For example, educators were observed reassuring their groups “I’ll rely on you for clinical knowledge” (Observation, Site 7) maintaining a distinction between educators’ own domain of expertise (the professionalism guidance) and that of the doctors.

Trustworthiness was also developed through relationship-building and personal rapport with the participants. For example, when describing a case where a doctor got into trouble, one educator said “I don’t want anything like that to happen to my doctors” (Observation, Site 1). Another educator referred to past cohorts throughout as “one of my” doctors or “some of my” doctors (Observation, Site 3).

Trust was further enhanced by educators acknowledging and permitting discussion of doctors’ negative perceptions of the regulator without becoming defensive, and the openness of educators to discussion and debate in general also impacted positively on participants’ perceptions of their trustworthiness:

P1: I think [the educator] has got good people skills and she’s able to communicate and she doesn’t talk at you; she talks to you. She’s a great listener…There’s a whole personality. She’s the right person for doing the job…and when I throw arguments at her, she was not becoming defensive.

P2: I just feel like for me she really sold the GMC to me! To me, she comes across as very genuine, very trustworthy.

P1: Foundation doctor/UK graduate/Site 3.

P2: Foundation doctor/UK graduate/Site 3.

The educators were therefore a key component of the intervention.

### The message (content)

Analysis revealed that four key elements of the PCT ‘message’ component were influential in changing professional attitudes: the nature of the appeal (rational and emotional); the use of evidence; an emphasis on reducing risk; and guidance as to how to reduce risk.

Qualitative and quantitative evidence was presented in order to make rational appeals to doctors about their professionalism. Qualitative results from a survey, in which patients were asked about the qualities they like to see in a doctor, were presented in the form of a word cloud that highlighted the importance placed on listening and communication. Case law was also used in making narrative, rational appeals. For example, the Montgomery vs. Lanarkshire medico-legal case [39] was used to illustrate points made about specific areas of guidance.

The educators referred to relevant sections of the guidance throughout the sessions observed. Although occasionally a participant questioned some guidance, for the most part the guidance was apparently accepted, and was treated as the best source of advice for doctors when in a difficult situation. For example, one educator told the group that it is “helpful” to refer to the guidance (Observation, Site 1). Another said “I honestly do believe it can be supportive for you” (Observation, Site 5). The perceived utility of having clear, rational guidance on professional behaviours was echoed by doctors:

It’s the standard advice from the regulator, so if you follow it, it can’t be wrong. And it’s good to have that framework. I think it is so much more clearly written now than it was.

GP-Consultants/UK graduate/Site 6.

Quantitative evidence was also used to articulate rational appeals. Several educators showed slides containing results from the National Training Survey [40], with the results drawn specifically from the Trust or hospital where the session took place in order to demonstrate that doctors’ professional attitudes needed to change. Quantitative evidence also included data about the regulator’s handling of complaints. This was used to make the point that the regulator’s response to complaints was proportionate, and that only a small number of doctors - those whose behaviour clearly contradicts the published guidance - get into serious professional difficulty:

And seeing like the breakdown of the yearly cases, so many have been brought to the GMC, and this many were thrown out, and that many went through to initial hearing, and that many... So just a sort of understanding of that process as well as, if you got a referral to the GMC, what happens. It’s not the case that everything goes straight to the Tribunal, because that is what you take home, fitness to practice and the stresses it involves.

Foundation doctor/UK graduate/Site 3.

This was used to make the rational argument that adhering to the regulator’s professional guidance is protective, however reference to complaints and serious professional consequences is likely to have elicited an emotional response amongst doctors as well.

The most prevalent emotional appeal was linked to fear, which was often framed in terms of risk to the doctors from behaving unprofessionally, but also included acknowledging other fears of doctors when faced with professional dilemmas. In a session covering raising concerns, an educator asked the group what the barriers to raising concerns might be. Suggestions from the group included a lack of response from the Trust, and detrimental outcomes for the doctor. The educator showed a word cloud about what doctors have said about raising concerns; these included “career-ending,” “fear,” “culture,” “seen as trouble-maker” and “no protection” (Observation, Site 5). The doctors’ potential worries about raising concerns were thereby acknowledged and accepted by the educator.

There were also emotional appeals relating to the doctors’ wellbeing. In the context of complaints relating to doctors’ health, an educator told their group that “you tend to think you’re superhuman” (Observation, Site 4). The same educator also acknowledged the increased risk of suicide that doctors face when being investigated by the regulator, adding that “You’re at a higher risk anyway aren’t you”. In both instances the educator was open about the pressures that doctors face, with this reference to emotion appealing to the group to emphasise the ‘human’ side of the regulator. It was also used to reiterate the point that adherence to professional guidance could reduce risk, and therefore fear.

Educators also provided practical suggestions as to how participants could mitigate risk. For example, one educator recommended keeping clear records for doctors’ own protection: “things that will really protect you: document and seek advice from the GMC” (Observation, Site 7).

The educators also spent time raising awareness of various resources that could be useful to the doctors. These included:

- Flowcharts of processes to follow e.g. when deciding whether confidentiality needs to be breached
- Mobile device applications
- Online resources provided by the regulator

This was done by describing the resources and the benefits of using them, and by showing images and screenshots of the resources on their slides.

### The audience (participants)

Observations revealed that the content of the sessions was highly relevant to the participants and was tailored according to their needs. Participants were engaged actively, using questioning and case-based discussion with peers, with educators giving participants the opportunity to contribute to plenary discussions and debriefs.

Message discrepancy - misalignment between the attitudes or knowledge currently held by participants compared with the educator’s message - appeared to be influential in changing knowledge and attitudes. For example, participants had variable understandings about the GMC’s professionalism guidance. The programme impacted mostly on international medical graduates and senior doctors for whom the course offered an opportunity to learn about current professionalism guidance as well as the UK context. Foundation doctors who had recently completed undergraduate education and taken the Situational Judgement Test (SJT) had more contemporary knowledge of regulatory guidance and thus less misalignment.

A lot of it we did have to revise and go through before the SJT and as part of medical school as well…it’s just reinforcing information.

Foundation doctor/UK graduate/Site 3.

High levels of message discrepancy were also demonstrated by participants in relation to how the regulator dealt with complaints. Most participants displayed negative attitudes about the regulator’s handling of complaints, and through engagement with the programme participants’ attitudes shifted to become more positive:

P1: Nowadays, I feel that they’re more transparent and approachable. Earlier, GMC means something like a disciplinary body, where they organise these workshops and they are coming to us, basically.

P2: I agree with my colleague. In the past, it looks like just… the GMC is a stick in the hand behind my back and not supporting, and a lot of information… GMC was all, stay away from the GMC, don’t do anything, otherwise you will be reported to GMC and the GMC will deregister you, your livelihood can be finished off. And after taking that… a bit more clear on how the GMC is looking towards doctors, what they expect from the doctors, so more of a… yes, patients is the main aim, as well as a human factor also, GMC also considers the human factor.

P1: Specialty doctor/International Medical Graduate/Site 2.

**P2: Specialty doctor/International Medical Graduate/Site 2**

I think my attitude is more favourable towards the GMC, particularly because [the educator] explained to us the very small proportion of cases that actually lead to major disciplinary action or dismissal or charges, and that most of what they do is, kind of, very supportive and, you know, they try to help doctors in difficulty or people who’ve had negative experiences. So, I think it’s improved my impression of the GMC.

Consultant-senior trainee /UK graduate/Site 1.

Thus, the programme appeared to lead to improved attitudes towards the regulator and their professionalism guidance.

## Discussion

A significant finding of our research was that, viewed through the lens of PCT, attributes of the educator were seen to be a key mechanism for influencing participants’ professional attitudes. These were principally: source credibility and trustworthiness. The educators’ credibility comprised expertise - demonstrated by knowledge of the professional guidance and the participants’ clinical context - and trustworthiness. Trust was established by: creating a space where participants were able to engage in debate and articulate their negative attitudes; deliberately establishing source similarity, emphasising shared experiences and real-world understanding; and using skilled communication techniques to develop rapport with participants.

Another significant finding was that the message directed to participants was seen to exhibit important attributes of persuasiveness: the nature of the appeal (rational and emotional); the use of evidence; an emphasis on reducing risk; and guidance as to how to reduce risk. Rational appeals included using qualitative and quantitative data, using logical argument and presenting data regarding the outcomes of complaints which framed the regulator’s response as proportionate. There were also emotional appeals; fear was sometimes used as a mechanism to promote compliance with published professional guidance. This message emphasised the GMC’s role in protecting doctors, and how by adhering to professional guidance, participants were at lower risk of adverse outcomes.

Finally, another important mechanism that influenced participants’ attitudes was message discrepancy. Following the workshops, participants showed a greater awareness and appreciation of the GMC’s role and their professional guidance, and some of those whose attitude was previously negative articulated a more positive view. Interactivity promoted active involvement of doctors with the teaching material and ensured that real world experiences were considered.

In the literature, it has been argued that class-room based approaches to teaching professionalism are misaligned with the nature of professional practice. Authors such as Gill & Griffin [16], Holtman [41] and Buyx et al. [42] highlight the socio-cultural nature of learning to be a professional and, consequently, calls for role modelling and other workplace-based approaches to play a dominant role in professionalism education are common [43, 44]. However, the real-world clinical context presents challenges to the implementation of professionalism in practice [45] and so there is an argument for appropriate classroom-based approaches to run alongside workplace experience in order to ensure that the correct messages, such as updated professionalism guidelines, are received by clinicians. As Boud et al. [46] observe, while experience is often taken to be the foundation of professional learning, it may not always lead to it.

Within the medical professionalism literature there are calls to develop better informed classroom-based approaches to teaching professionalism [47–49] and to develop educational approaches that take account of professional experience [50]. Our study addresses this gap by means of an examination of a classroom-based professionalism education intervention, explored through the lens of PCT.

Our study revealed that the way educators conduct the educational intervention is important. Pedagogically, the interactive approaches observed within the programme served the purpose of surfacing what Wynia et al (p. 714) [51] describe as doctors’ normative beliefs about professionalism, exposing these to public scrutiny and debate. This aligns with Karnieli-Miller et al.’s [52] finding that interpersonal interactions with faculty and peers are important influences in shaping the professional attitudes of learners, as well as allowing doctors to explore the application of ‘practical wisdom’ that Hilton and Southgate (p. 267) [53] argue is at the heart of medical professionalism. In a field in which there is room for improvement in teaching [54], and in which there is often disagreement about how professionalism might best be taught [55], our research suggests that classroom-based approaches, understood as persuasive communication interactions, can provide a valid, effective way to influence doctors’ professional attitudes.

### PCT as an approach to researching professionalism education

Since it was first proposed by Hovland and colleagues as a way of understanding attitudinal change [56] PCT has been widely used as a theoretical framework underpinning attitudinal research in the domains of marketing and politics [12] - fields that are intuitively linked with the act of persuasion. To a lesser extent, PCT has been used to understand public health interventions, for example in HIV [27] and smoking-related campaigns [26]. However, despite Hovland’s description of persuasive communication as a learning theory [56], the use of PCT as an underpinning theoretical framework in education research has been extremely limited, being largely confined to a small number of studies exploring changes in schoolteacher and student attitudes to mathematics and science education [57, 58, 59]. As previously described, there is no literature evaluating the utility of PCT for conducting attitudinal research in professionalism education in medicine.

According to Shrigley and Koballa [60] the largely non-theoretical treatment of attitudinal research in education is the principal reason for the apparent lack of progress within the discipline over a 20 year period. Similarly, researchers in social psychology have described non-theoretically driven attitudinal research as being ‘chaotic and confusing’ (ibid., p. 19). It is therefore important that research into changing professional attitudes in medical education does not fall into the same trap. Rather, researchers should adopt and articulate a clear conceptual framework that can function as a theoretical net to draw together observations that would otherwise be disorganised and difficult to interpret. In our view, PCT offers a compelling theoretical framework for doing so.

### Strengths and limitations of the research

A particular strength of the research undertaken for this study was the robust nature of the methodology, which addressed key elements of quality and dependability in qualitative research [61]. Thus, our research methods comprised: a multi-methods exploration of the phenomenon; maximum variation sampling; multiple iterations of coding, discussion between researchers and re-coding; and the use of a conceptual framework that afforded the ability to explore persuasive communicative acts theoretically. As such, it offered a lens through which to view the tensions and important interrelationships between different elements of the persuasive communication process. A limitation of our study was that our conclusions about the effectiveness of the approach in terms of attitudinal change had to be inferred from the data. A further limitation was that the sample population comprised doctors who had volunteered to participate in the GMC’s Duties of a Doctor programme, and thus were arguably pre-disposed to value, and be persuaded by, the educators’ input.

## Conclusion

This study has extended the medical education literature by providing a theoretical understanding of a professionalism education programme using the lens of persuasive communication. In doing so, we found that a classroom-based intervention that runs alongside and acknowledges professional experience can have a positive impact on doctors’ professional attitudes if it adheres to known features of persuasive communication. Persuasive communication theory has not previously been widely adopted within the field of professionalism education research in medicine, despite its core premise linking communication to attitudinal change. This study provides an insightful appreciation of the mechanisms of a national professionalism education intervention so that educationalists can look to maximise the effectiveness of local professionalism education programmes.

## Data Availability

The data underpinning this study are not available as consent for this has not been granted by participants.

## Abbreviations

GMC: General Medical Council
GP: General practitioner
PCT: Persuasive communication theory
SJT: Situational judgement test
